# Atlanta Academy of Medicine

**Published:** 1874-05

**Authors:** James B. Baird, A. W. Calhoun

**Affiliations:** Reporter; Reporter


### Atlanta Academy of Medicine.

JAMES B. BAIRD, M.D., REPORTER.

A. W. CALHOUN, M.D., REPORTER.

ATLANTA, March 9, 1874.

Dr. W. S. Armstrong, President, presiding.

Dr. J. M. Johnson reported the case of a lady, who, after two years cessation of the menstrual periods, was seized with a severe hemorrhage, which very much exhausted her. In a few days thereafter, she was taken with violent pains in the back of the head and neck, extending a short distance down the spinal column. The pulse was weak and slow, continuing so as long as the pain remained so intense. By the use of cupping and purgatives, the symptoms grew better in a short time. A few nights afterwards, as a band of music began playing in the immediate vicinity, she sprang suddenly from bed, with incoherent exclamations, and fell back in an insensible condition. He found her suffering from a circumscribed pain in the back of the head, the left eye lid closed and the ball drawn far to the outerside, the pupil widely dilated aud unable to see with the left eye. The pain was relieved by injections of black drop by the rectum, and the hypodermic use of morphine. Patient has improved since the second attack, but the eyelids and ball remain the same. Up to the moment when the music began, she was free from pain.

Dr. Johnson asked what is this trouble about the eye, and how produced?

Dr. J. G. Westmoreland thought the case to be one of neuralgia. In debilitated conditions we not unfrequently have symptoms in neuralgia similar to those just given.

Dr. J. M. Johnson, in reply to Dr. Taliaferro's inquiry as to her previous health, said it had been excellent up to the time of the hemorrhage, for the two years preceding. Drs. Miller and Cumming, who were called in consultation, agreed with him, that it was a case of meningitis. Most of the serious symptoms had disappeared previous to the night when the band of music began playing in the neighborhood, followed by the results narrated above. If effusion took place, how is it to be removed, and is it safe for the patient to travel?

Dr. Taliaferro thinks the uterine hemorrhage possesses pathological significancy, and suggests that the pain in the back of the head and neck, is the result of reflex irritation, and that the present trouble with the eye could be thus accounted for.

Dr. DeWitt asked if effusion did occur, could it not have also produced rupture of the nerve supplying the muscles of the eye and the consequent troubles.

Dr. Love requested to know if the excessive hemorrhage was uterine. Gave an anatomical description of the different nerves supplying the different muscles of the eye, and the effect produced, when each or all became paralyzed.

Dr. W. F. Westmoreland thought the woman was laboring under hysterical paralysis; had seen hardly perceptible uterine affections, complicated with severe troubles about the eye, paralysis of the vocal cords, etc.

Dr. Calhoun agreed fully with Dr. Johnson, that the levator of upper lid and the muscles of the eye ball, with the exception of the external rectus and superior oblique had lost their power by paralysis of the third pair of nerves (the motor oculi,) which was caused by an effusion of some kind at the base of the brain, pressing upon the nerve at its origin, or upon some point in its course, or possibly the effusion might have taken place within the sheath of the nerve. A small branch from the third pair is given off to the external rectus, which accounts for the partial implication of this muscle in complete paralysis of the third pair. The main nervous supply of the external rectus is received from the fourth pair, that of the superior oblique from the sixth pair of nerves. The dilatation of the pupil and the impairment of vision depended, no doubt, upon the paralysis of the muscular fibres of the iris and of the muscle of accommodation, the ciliary muscle.

In this case the probability was, that the inability to see, was for near objects, whereas, for distant objects the vision was as good as formerly. If meningitis often left the auditory and optic condition mentioned, because of pressure from effusions, etc., why could not the same hold good as regards the other nerves supplying the muscles of the eye? Recommended constitutional treatment and the application of large blisters behind the corresponding ear, with the local use of electricity.

Dr. J. M. Johnson thought it was time to cease to impose all the unaccountable ailments of the female body upon the uterus. Ordinarily it is a very modest organ, and unable to bear such a burden. In this particular instance, thought the hemorrhage came from within the uterus without disease. Considered the case primarily to be one of meningitis, and was sustained in his diagnosis by the consulting physicians, and that the affection of the eye was attributable to this disease.

Dr. Taliferro remarked that where menstruation had ceased for two years, the uterus usually becomes atrophied, and in a case of this kind would fear some severe uterine lesion.

---

Atlanta, March 30, 1874.

Dr. W. S. Armstrong, President, presiding.

Dr. Wilhite, of Anderson, S. C., being present, reported a case of diabetes mellitus of four years standing; a man æt. forty. For two years passed six gallons of urine in twenty-four hours; the amount is now reduced to about two and a half gallons a day. He is sometimes so much reduced that death is threatened for a week or two. He then improves and recovers sufficiently to be up and walking about. A great variety of remedies has been tried, and if benefit has been derived from any, it is from ergot and tannin. As soon, however, as the urine is reduced anasarca comes on. Notwithstanding this enormous discharge by the kidneys, he sometimes has as many as thirty actions on his bowels in the twenty-four hours. An examination of the urine shows sugar in abundance.

Dr. DuPre had read an account of a case in which the sugar diet was resorted to with success. He recommended in this case a trial of creosote.

Dr. Battey suggested, as the pathological lesion is situated in the nervous system, that a trial of bromide of potassium be made sufficiently to fully impress the nervous system. To test its efficacy, one hundred and twenty grains might be given in the twenty-four hours, for some days.

Dr. DeWitt proposed brewers' yeast as calculated to hasten the digestive process. Had seen this remedy used with apparent benefit, when a student of medicine in Philadelphia.

Dr. Jno. G. Westmoreland called the attention of members to the subject of phlegmasia dolens or milk leg, and asked for the best mode of treatment. There are two theories of the pathological condition; one holds that it is an inflammation of the veins, the other ascribes the affection to the nervous system. He requested the opinion and experience of the Academy.

Dr. Logan, during the past week, had seen a lady who was delivered about two weeks before; she presented serious symptoms of milk leg. Pain and swelling particularly in the calf of the leg. He thinks the weight of evidence is in favor of subacute phlebitis, dependant upon uterine irriatation. In this case, the treatment consisted in the application of an ointment of iodine and carbolic acid to the leg, and calomel and hyoscyamus internally. His object was to allay the irritability of the uterus. Recovery was rapid.

Dr. Armstrong has met with three cases during the winter. He considers it a phlebitis. None of the cases referred to were marked by great constitutional disturbance. The leg was painful, the femoral vein was tender and hard throughout its course; this condition in one case, extended to the veins of the foot. In another the veins running up over the abdomen were similarly affected, presenting a cord-like feel. Leeches were applied along the course of the femoral vein; the leg was enveloped in hot dry cloth and a cathartic administered in each case. In two cases the unpleasant symptoms vanished in three days. In one they continued for ten days. This condition supervened immediately upon parturition. Last Monday, one week ago, was called to see a lady in the third month of pregnancy. She stated that for two days she had had slight uterine hemorrhage without pain. Diagnosis, threatened abortion. Ordered her to keep her bed, and prescribed fluid extract viburnum prunifolium, and bromide of potassium. She had uterine pain and hemorrhage on Wednesday. When he arrived, he found the vagina filled with clot, pains feeble, os open; ordered ergot. In an hour or two the os was closed and the hemorrhage had ceased. Two days later she experienced pains; found the os dilated and the membranes partially protruding, no hemorrhage; gave ergot with no effect. This condition continues; to-day a somewhat putrid odor was observed. She complained of pain in the back and has some fever. An effort to remove the membranes by forceps was made, after failing with the finger, but their structure was so fragile that nothing was accomplished, and the attempt was not renewed.

Dr. Logan always in such cases tampons; fears hemorrhage; is not satisfied until the contents are discharged.

Dr. Battey regards no woman in this condition absolutely safe for an hour. A case was recently reported to the Academy illustrating this point. Advised that the uterus be pressed down into the pelvic cavity, and that the index finger, used as a blunt hook, be pushed beyond the mass, and a rotary motion be given to it at the time traction is used; considers it bad practice to allow such a mass to remain in the uterus and become putrid. If the contraction of the os cannot be overcome by the finger, which he much prefers, advises the use of a tent for that purpose. In case of failure the vagina should be tamponned. The presence of the tampon favors the expulsion of the uterine contents.

Dr. Jno. M. Boring's experience in cases of abortion corroborates the foregoing. His rule is, if the finger can pass the os, never to leave his patient until the contents of the uterus are expelled. If the os is not dilatable he tampons, and in nine out of ten cases the contents come away when the tampon is removed.

Dr. Armstrong added that this was the most rigid os he had ever seen. Prolonged, but unsuccessful attempts were made to remove the mass, which is pear shaped, having a broad base corresponding to the fundus uteri. Asked the opinion of the Academy as to the propriety of introducing forceps and breaking down the mass.

Dr. Battey condemned the suggestion. It would simply convert one into many masses. Entertains decided objections to all placental forceps.

Dr. J. G. Westmoreland saw no objection to breaking up the mass. Thought if it was divided that it might be expelled by the forces now existing.

Dr. Taliaferro thought the course to pursue in this case depended upon the existence or non-existence of complications.

If there is no tenderness in or around the uterus, remove the mass. If there is, should be afraid to use any manipulation, but place the woman on her back and give opiates. There is no danger; the contents will escape by liquifaction. Is always loth to interfere if tenderness exists in the roof of the pelvis.

Dr. DuPre suggested a tampon of soft material medicated with starch, carbolic acid and opium. Thinks it safe to let alone. Decomposition in such cases is frequently restricted to such portions of the uterine contents as are exposed to the air. Would fear metritis if active interference was resorted to.

